# Telehealth in a paediatric developmental metropolitan assessment clinic: Perspectives and experiences of families and clinicians

**DOI:** 10.1111/hex.13582

**Published:** 2022-08-17

**Authors:** Chan Esther, Ong Natalie, Barnett Diana, Hodge Marie Antoinette, Drevensek Suzi, Williamsz Marcia, Silove Natalie

**Affiliations:** ^1^ Child Development Unit The Children's Hospital at Westmead Westmead New South Wales Australia; ^2^ University of Sydney Sydney New South Wales Australia

**Keywords:** developmental disorders, diagnosis, paediatric service, telehealth assessment

## Abstract

**Background:**

The application of telehealth in the paediatric setting is growing, and yet, limited research has focused on using telehealth in developmental diagnostic assessment and the consumers' perceptions of their telehealth experience. This study explored parents'/carers' and staff experiences of using telehealth as part of the developmental diagnostic assessment.

**Methods:**

Parents/carers who attended an assessment between June 2020 and July 2021 that incorporated a telehealth component within a hybrid service delivery model were invited to provide feedback about their experience of telehealth appointments at a multidisciplinary developmental assessment service. All parents were invited to complete an online survey, with a sample of families being offered a telephone interview. Staff members were invited to a focus group to explore their experiences of delivering services via telehealth. Data obtained were analysed descriptively and thematically using a mixed method of analysis. Codes were categorized, enabling facilitators and barriers to be explored.

**Results:**

The use of telehealth in the diagnostic assessment of complex developmental disorders received high levels of acceptance from parents/carers and staff, despite having limitations such as technical issues, difficulties building rapport between families/clinicians and limited direct observations of the child. Telehealth services are perceived to reduce costs and increase flexibility, including increased ability to accommodate family needs.

**Conclusions:**

Results demonstrated that telehealth is a highly acceptable mode of service in a developmental assessment service. The current study informs the development of a hybrid service delivery model by enhancing facilitators and reducing barriers commonly reported by consumers and provides direction for future research.

**Patient or Public Contribution:**

Parents or carers of children who attended a tertiary paediatric assessment unit for a diagnostic developmental assessment completed the online survey and were interviewed.

Since the COVID‐19 pandemic began, telehealth has gained increased attention due to public health restrictions imposed on face‐to‐face interactions.^1^ Previous studies in the paediatric population have shown that telehealth reduces time and financial costs to families and improves communication between healthcare service providers along with improving access to care.^2^, ^3^, ^4^ Telehealth has the potential to minimize disruptions to service provision and wait times while protecting clinicians, families and children.

## 1 TELEHEALTH IN PAEDIATRIC OUTPATIENT CONSULTATIONS

There has been increase in research examining the acceptability and effectiveness of telehealth in the paediatric healthcare setting. Many paediatric outpatient clinics, for example, ophthalmology, orthopaedics and pre‐ and postoperative clinics, include history taking and clinical interview as part of their service. Though ordinarily conducted face to face, this can be easily delivered via telehealth. Telehealth consultations have been found to be highly accepted by families and clinicians for outpatient appointments in many countries such as Australia, Canada, the United Kingdom and the United States.^3^, ^5^, ^6^, ^7^, ^8^, ^9^, ^10^, ^11^

Patients, clinicians and referrers of paediatric outpatient services have expressed a willingness to use telehealth, with the advantages relating to convenience and cost savings.^4^, ^9^ However, the potential technical difficulties, concerns about child participation in the telehealth session, lack of physical interaction and preference for face‐to‐face appointments were reported to be the main barriers to telehealth uptake.^4^, ^12^, ^13^, ^14^, ^15^ Due to the circumstances surrounding COVID‐19, there has been an increased willingness to use telehealth,^2^ recognizing the importance of balancing the need for accessing services while adhering to physical distancing guidelines. There is a need to address these impediments, which, if mitigated, could increase the ability of clinical services to be delivered via telehealth in the context of increasing acceptability by families and clinicians.

## 2 TELEHEALTH DIAGNOSTIC ASSESSMENT OF DEVELOPMENTAL DISORDERS

Diagnostic assessments for children for whom there are developmental concerns have traditionally been conducted in the face‐to‐face setting and can take many hours to complete. Such assessments require expertise and utilize a multidisciplinary approach to understand the complex and dynamic nature of the child–environment interaction on development. The challenge of using telehealth in diagnosing developmental disabilities is manyfold. Issues specific to telehealth diagnostic processes pertain to missing out nuances of child responses, not being able to completely assess parental psychological and emotional states and not being able to directly play and interact, as well as interruptions due to connectivity and technical glitches.^16^

Evidence on the use of telehealth for diagnostic and developmental assessments is slowly emerging, but still limited. Research has shown that diagnostic interviews can be conducted via telehealth such as The Autism Diagnostic Interview‐Revised and Developmental, Dimensional and Diagnostic Interview.^17^, ^18^ Novel telehealth diagnostic assessments of autism spectrum disorder (ASD), including the Brief Observation of Symptoms of Autism, Tele‐ASD‐Peds and the Naturalistic Observation Diagnostic Assessment, have been developed and demonstrate promise through preliminary findings.^19^, ^20^, ^21^, ^22^ Telehealth‐delivered standardized cognitive and language assessments for children have demonstrated high fidelity, reliability and acceptability.^15^, ^23^, ^24^ However, further work is needed to understand parents' and clinicians' perceptions and confidence in using telehealth within the clinical setting.

## 3 DEVELOPMENT OF A HYBRID SERVICE MODEL

The onset of the COVID‐19 pandemic brought a great deal of uncertainty and difficulty in predicting the return of face‐to‐face assessments. Consequently, many paediatric developmental diagnostic services have been faced with increasing waitlists of children with developmental, behavioural and psychosocial issues. It has become apparent that to maintain service delivery, new approaches are needed.

**Figure 1:**
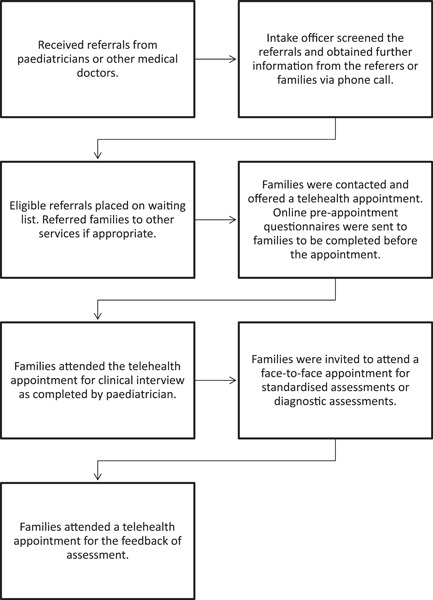
Service delivery model.

The current study was conducted within a tertiary paediatric developmental assessment unit covering the Western Sydney Local Health District and offering a statewide service for children who do not have access to a local developmental assessment service within New South Wales, Australia. As this unit has contributed significantly to telehealth research in assessments of children^15^, ^23^, ^24^ with language and learning difficulties predating COVID‐19, it has existing infrastructure including technology and trained staff accustomed to the use of telehealth. This meant that during the first COVID‐19 lockdown in Australia, the service was able to shift service delivery from face‐to‐face clinics to telehealth. With restrictions imposed on face‐to‐face clinical appointments, the team planned to replace face‐to‐face with telehealth‐only consultations for 3 months (from April to June 2020), with a view to an additional face‐to‐face appointment when restrictions eased. From July 2020, the unit commenced a hybrid clinic model (see Figure 1) developed as a planned response to the pandemic restrictions. Face‐to‐face appointments were conducted in accordance with the public health and hospital regulations around wearing masks, hand sanitization, sanitization of equipment and limiting the number of people per room, as well as reducing assessment duration to a maximum of 2 hours.

## 4 CURRENT STUDY

Quality improvement initiatives have long been used for the ascertainment of the efficacy, reliability and safety of health services. As telehealth appointments were introduced with all families before face‐to‐face assessment, it was critical to ascertain the acceptability from the perspective of the participants. This project, therefore, aimed to obtain the perspectives of families and staff members regarding the use of telehealth as part of the service model within a developmental assessment clinic.

## 5 METHODS

### 5.1 The Plan Do Study Act cycle

**Figure 2:**
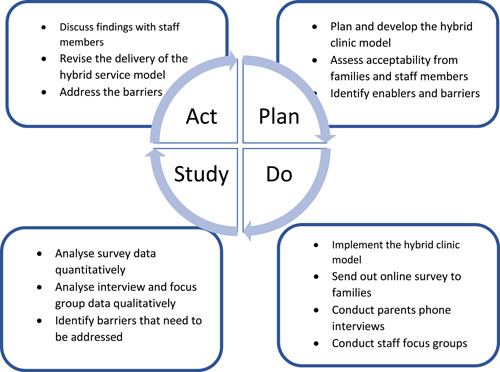
Plan, Do, Study, Act cycle.

To inform future service development, evaluation using the Plan Do Study Act (PDSA) cycle (see Figure 2) was implemented between 14 May 2021 and 15 July 2021.

#### 5.1.1 Plan

The unit developed a hybrid service delivery model in response to public health restrictions imposed on face‐to‐face interactions. This study was submitted and approved as a Quality Improvement project by the relevant Clinical Governance Unit (QIE‐2021‐02‐25) to assess the acceptability of the new clinic model and identify enablers and barriers.

The parent/carer survey was developed by the Sydney Children's Hospitals Network to study the experiences and acceptance of patients/families using telehealth in outpatient clinics across the COVID‐19 period. The survey contained 24 questions and focused on 5 key areas: the ease of use, usefulness, patient/family experience, technical quality and usage intention. Multiple‐choice questions and Likert scales were used in the survey.

#### 5.1.2 Do

The unit implemented the hybrid service delivery model from July 2020. The hybrid clinic adopted by the unit was conducted over 2–3 separate occasions of service as follows:

1. Conducting multidisciplinary team clinical interview via telehealth. The Vineland Adaptive Behaviour Scales, Third Edition (Vineland‐3),^25^ was also administered if suitable.
2. Contacting school or preschool and allied health professionals between telehealth and face‐to‐face appointments.
3. Partial completion of the report before the face‐to‐face assessment.
4. Providing time‐limited, face‐to‐face standardized assessments such as the Wechsler Intelligence Scales for Children–Fifth Edition,^26^ the Wechsler Preschool and Primary Scale of Intelligence—Fourth Edition,^27^ Mullen Scales of Early Learning,^28^ Stanford–Binet Scales of Intelligence‐Fifth Edition,^29^ the Wechsler Adult Intelligence Scale‐Fourth Edition,^30^ the Wechsler Individual Achievement Test‐Third Edition^31^ and the Autism Diagnostic Observation Schedule‐Second Edition.^32^
5. Feedback of assessment outcome to families via telehealth or face to face.

Parents/carers of children aged between 0 and 18 years, who had a telehealth appointment as part of the diagnostic assessment within the unit between June 2020 and July 2021, were given information about the evaluation study. Towards the end of the assessment, verbal consent was obtained from parents/carers to email them the link to the survey. Participation in the survey was optional and anonymous. In addition, parents/carers not requiring an interpreter were asked if they were willing to be contacted by authors D. B. and E. C. at a later stage for a telephone interview to gain further feedback about the hybrid model of care. The interviews were approximately 10–15 min each. All interviews were voice‐recorded and followed a script (Table 1), which were then transcribed verbatim. All identifiable information was removed at transcription. Demographic information regarding the children and families who completed the survey is shown in Table 2.

**Table 1:** Semi‐structured interview with parents/carers

Questions
1 Can you tell us about your recent telehealth experience when having your child assessed through the unit?
2 What did you think about the experience?
3 What did you like about the telehealth experience?
4 What did you not like about the telehealth experience?
5 How can we improve our telehealth and clinical services further?
6 In future, would you prefer telehealth or in‐person appointments? Are you able to tell us why?

**Table 2:** Demographic information of families who completed the survey

Variables	*n* (%)
Age of the child	
0–5 years old	10 (40.0)
6–9 years old	8 (32.0)
10–12 years old	1 (4.0)
13–15 years old	4 (16.0)
16–17 years old	1 (4.0)
18 years old and older	1 (4.0)
Geographic location	
Urban (population above 100,000)	24 (96.0)
Regional/rural/remote (population below 100,000)	1 (4.0)

Staff members were also invited to provide their feedback and experience of the hybrid service delivery model in a focus group. The focus group was led by an independent facilitator who did not work within the unit. Before commencing, all participants (14 staff members in total, comprising 2 paediatricians, 1 paediatric registrar, 2 neuropsychologists, 2 speech pathologists, 2 social workers, 1 clinical nurse consultant, 2 occupational therapists and 2 administrative officers) received an information sheet about the focus group and completed a written consent form. The focus group was conducted over 2 days, with each session lasting 2 hours. The focus group was voice‐recorded for later transcription. All identifiable information was removed at transcription.

#### 5.1.3 Study

The data collected from the parents/carers survey were analysed descriptively. The parents'/carers' interviews and staff focus group feedback were analysed thematically using the Framework approach.^33^ After transcription, authors D. B. and E. C. familiarized and inductively coded the transcripts independently. It is noteworthy that authors D. B., E. C. and N. O. did not actively take part in the focus group. Authors D. B., E. C. and N. O. then subsequently discussed and reviewed the codes, before grouping them into categories. Consensus agreement was reached by all authors that saturation was reached.

#### 5.1.4 Act

The themes derived from the parents'/carers' interviews and staff focus group by using the Framework approach informed the enablers and barriers of integrating telehealth into the hybrid diagnostic assessment process. Service delivery improvements were implemented according to the themes, which are described in the discussion section.

## 6 RESULTS

### 6.1 Parents'/carers' evaluation survey

The online survey was sent to 61 parents/carers and 27 responses (44.3%) were received. Two surveys were excluded from the current study due to invalid responses, resulting in the inclusion of a total of 25 surveys in the analysis. Results indicated that 56.0% of parents/carers used telehealth for the first time (see Table 3). Only 12.0% of the telehealth appointments took more than 10 min to set up and three telehealth appointments were unsuccessful. Two telehealth appointments required switching telehealth platforms due to poor visual and audio quality.

**Table 3:** Online survey about the parents'/carers' feedback of telehealth appointment

Variables	*n* (%)
Device/s used for the telehealth session	
Iphone	6 (24.0)
Ipad	3 (12.0)
Android phone or tablet	9 (36.0)
Personal computer (laptop, desktop)	7 (28.0)
A phone for a telephone call	5 (20.0)
Telehealth platform	
PEXIP	11 (44.0)
Coviu	2 (8.0)
Zoom	1 (4.0)
Facetime	2 (8.0)
Phone call	5 (20.0)
I started on one platform, then needed to change to another	2 (8.0)
Unsure	2 (8.0)
Clinician/s involved in the telehealth session	
Doctor/s	13 (52.0)
Nurse/s	2 (8.0)
Psychologist/s	13 (52.0)
Social worker/s	7 (28.0)
Speech pathologist/s	7 (28.0)
Occupational therapist/s	4 (16.0)
General practitioner	1 (4.0)
Other	3 (12.0)
Types of appointments that parents/carers would be comfortable having via telehealth in the future	
First‐time appointment with a new department	6 (24.0)
First‐time appointment with a new clinician	6 (24.0)
Follow‐up appointments	17 (68.0)
Counselling/mental health appointments	8 (32.0)
Good news results	15 (60.0)
Bad news results	9 (36.0)
When a physical assessment is needed that can be completed over video	6 (24.0)
Any appointments when the clinicians say we don't need to be face to face	18 (72.0)
None, I would like all possible appointments to be face to face	1 (4.0)
Advantages for parents/carers in using telehealth instead of a face‐to‐face appointment	
Able to access specialist care that isn't available where I live	1 (4.0)
Receive advice to help understanding/manage the condition	2 (8.0)
Allow other members of my healthcare team to attend the consultation	6 (24.0)
Convenience	22 (88.0)
Save money	9 (36.0)
Save time (e.g., didn't take as much time off work/school)	19 (76.0)
Able to stay closer to home and/or family	12 (48.0)
Allow for social distancing/isolation	19 (76.0)
Disadvantages for parents/carers in using telehealth instead of a face‐to‐face appointment	
Not being physically present meant there were limitations to the consultation	8 (32.0)
Delay in receiving prescriptions or pathology/radiology requests	3 (12.0)
Unable to be given paper copies of documentation	3 (12.0)
Did not have easy access to internet‐enabled devices or Wi‐Fi/broadband	2 (8.0)
Issues with interpreter services	1 (4.0)
Poor video quality	1 (4.0)
Difficult to concentrate in telehealth session in home setting	1 (4.0)
There were no disadvantages	13 (52.0)
Technical issues during the telehealth session	
The sound was difficult to hear	7 (28.0)
The video was difficult to see	2 (8.0)
The connection dropped out unexpectedly	1 (4.0)
The equipment didn't work (e.g., microphone, webcam)	1 (4.0)
The clinician/s had technical difficulties on their side	3 (12.0)
Needed to change from one platform to another because the technology wasn't working	2 (8.0)
There were no issues	15 (60.0)

The majority of parents/carers agreed that they were involved in decisions about their child's care and treatment (92%), felt that their child was treated respectfully (96%) and all parents/carers reported that the clinicians explained things in a way they could understand. Furthermore, 64% of parents/carers believed that their children felt comfortable during the telehealth appointment, while 28% of them were unsure about how comfortable their children felt. Most parents/carers (92.0%) were happy with the service their children received.

Parents/carers perceived convenience, time saving and allowing for social distancing as the top three advantages of telehealth appointments. More than half (52%) of the respondents suggested that there were no disadvantages of having appointments via telehealth. Forty‐eight percent of parents/carers indicated that they were extremely likely to recommend telehealth to friends and family, with only 20% not so likely to recommend to others. However, 64% of parents or carers reported that they would use telehealth again and 12% were unsure.

### 6.2 Parents'/carers' semi‐structured interview

A random sample of 11 parents/carers participated in the semi‐structured interview (see Table 4). The age of the children ranged from 3 to 17 years (63.6% male). An overall positive experience was reported by families, even though some technical issues were present during the telehealth appointment: ‘I didn't really have anything negative besides when there are interruptions that you cannot help it’ (Child B). Families also commented that the staff were attentive, polite, thorough and respectful in the appointments: ‘Over the phone they were always respectful towards her, they listened to what we were saying’ (Child E).

**Table 4:** Demographic information of families who completed the phone interview

Child	Gender	Age	Languages spoken at home	Assessments completed	Diagnoses	Notes	Preference for appointment type
A	M	16 years and 1 month	English	WISC‐V, Vineland 3	ADHD, ID, ASD		Face‐to‐face
B	F	6 years and 4 months	English	WPPSI‐IV, ADOS‐2, Vineland 3	No diagnosis was given		Hybrid
C	M	5 years and 10 months	English, Telugu	WPPSI‐IV, ADOS‐2	ASD, Language Disorder	Born in South Asia	Hybrid
D	M	10 years and 8 months	English	WISC‐V, ADOS‐2, Vineland 3	ADHD, ASD		Face‐to‐face
E	F	3 years and 6 months	English	Mullen, ADOS‐2, Vineland 3, Preschool visit	GDD, Speech Sound Disorder		No preference
F	F	4 years and 8 months	English	SB‐5, Vineland 3	ASD, GDD, Speech Sound Disorder	Parents with Oceania ethnicity	Face‐to‐face
G	F	4 years and 3 months	English	Mullen, Vineland 3	ASD. GDD, Speech and Language Disorder	Parents with English as a second language	Hybrid
H	M	4 years and 3 months	English	Mullen, ADOS‐2	ASD, GDD, Language Disorder	Parents with English as a second language	Hybrid
I	M	17 years and 2 months	English	WAIS‐IV, WIAT‐III, ADOS‐2, Vineland 3	ADHD, ASD	Live in remote areas	Hybrid
J	M	4 years and 9 months	English, Telugu	WPPSI‐IV, ADOS‐2, Vineland 3	ASD, Mild ID, Language Disorder	Parents with English as a second language	Telehealth
K	M	3 years and 1 month	Mandarin, some English	Mullen, ADOS‐2, Vineland 3	ASD, Language Disorder	Parents with English as a second language	Face‐to‐face

Abbreviations: ADHD, attention‐deficit/hyperactivity disorder; ADOS‐2, Autism Diagnostic Observation Schedule‐Second Edition; ASD, Autism Spectrum Disorder; GDD, global developmental delay; ID, intellectual disability; Mullen, Mullen Scales of Early Learning; SB‐5, Stanford–Binet Scales of Intelligence‐Fifth Edition; WAIS‐IV, Wechsler Adult Intelligence Scale‐Fourth Edition; WIAT‐III, Wechsler Individual Achievement Test‐Third Edition; WISC‐V, Wechsler Intelligence Scales for Children–Fifth Edition; WPPSI‐IV, Wechsler Preschool and Primary Scale of Intelligence—Fourth Edition; Vineland 3, Vineland Adaptive Behaviour Scale‐Third Edition.

#### 6.2.1 Advantages of the split service delivery model

##### Convenience and flexibility

**Figure 3:**
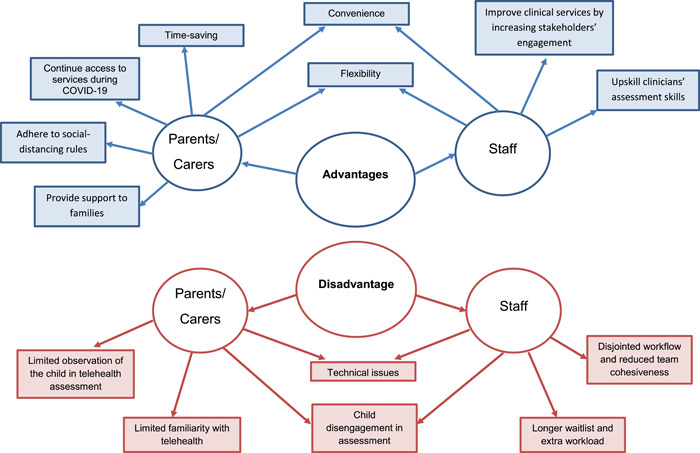
Themes of the hybrid service model emerging from parents'/carers' interview and staff focus group.

Families enjoyed the telehealth appointment for its convenience (see Figure 3), as indicated by their report of being able to attend the appointment from anywhere: ‘we can go back and do the work because it is online… It was easy to fit around work. I don't need to take leave and my husband to be on that day’ (Child J). Families can save travel time to the hospital, be more flexible with their time and reduce financial cost: ‘We did not have to travel because (the appointment was) on a working day. I just had to take the time out… otherwise I have to plan for it and take another 2 or 3 h (off work and spend) 40 min there and 40 back and 1 h at the hospital. It did save commute time’ (Child C); ‘I think it was very efficient time wise. Both of us could be there to the hour, there is no travelling time and it was cheaper’ (Child I).

##### Access to service during the pandemic

Parents/carers valued the telehealth appointment because it continued to provide access to services amid physical distancing, lockdown and geographical distance: ‘Obviously due to COVID it was helpful. We didn't have to go out in the community and put ourselves at risk. Yes, so that was definitely helpful’ (Child E).

##### Support to families

Parents/carers indicated that they were being supported by clinicians during the telehealth appointments: ‘I wrote a lot of the stuff down like the questions we wanted to get answered and they answered everything we needed to know and put us in the direction that we needed to go’ (Child F). Parents/carers also expressed that the hybrid service delivery model during the pandemic allowed for the initiation of the assessment process and access to early intervention to commence before a face‐to‐face appointment: ‘It is a chance to clear the air or his progress and where he is up to and doctor explained… therapies needed to be done. It was clearly explained about my situation in both my child and everything went well with telehealth at home’ (Child H).

#### 6.2.2 Disadvantages of the split service delivery model

##### Technical issues

Poor internet connectivity, audio issues and glitches on telehealth platforms were reported to be the common challenges that parents/carers encountered during the telehealth appointment: ‘The platform was difficult to get into’ (Child A); ‘There were a few hiccups with the freezing of the screen and things like that here and there but (the clinician) picked up where we left off when it came back to normal again during the time’ (Child B).

##### Disengagement in telehealth appointment

Children's attention was reported to vary, with some being more distractible when attending this style of appointment, while others were more focused: ‘I think (the clinician) can get a much better understanding of my child in particular from being in person rather than on a computer on a platform… he doesn't know the difference between the video call right at the end of the day but he video calls his father twice a day… and to him it's a game, it wasn't serious’ (Child A); ‘We are in a safe environment and they are comfortable and they are focussed on the screen’ (Child B).

##### Limited direct observation of the child

Parents/carers expressed concerns about completing the assessment with their child using only telehealth. They believed that the clinicians are not able to complete all assessments via telehealth or to fully observe and understand their child when observed through the computer: ‘I feel (the clinician) can't really assess, face to face is better so they can really assess my child’ (Child H).

##### Limited experience of using telehealth

Unfamiliarity with the telehealth platform and technology was also reported to be a challenge for this hybrid service delivery model. Some families had limited experience in using the technology: ‘I think there are people that are not very tech. It might be a bit difficult to get on line and log on and do’ (Child B).

#### 6.2.3 Preference of appointment type

Overall, despite the convenience, flexibility and opportunity to access the service during a pandemic, face‐to‐face appointments were perceived by parents/carers as being preferable over telehealth for engaging their child to gain a full understanding of their complex presentation: ‘For my daughter, I don't speak for other family, she can't really talk much because she can say some words she doesn't know how to talk on the phone you need to see her in person’ (Child G). The personal interaction that occurs in the context of in‐person appointments was also seen as preferable: ‘I think all in all I would prefer the in person even though it saves me a drive down there, I like to look at somebody in the eye and be in the same room when I talk to them as much as possible’ (Child D).

Appointments for clinical interviews, assessment feedback and any follow‐up sessions that did not involve direct assessment of the child were perceived as being more suited to telehealth: ‘If you just want to know what is the feedback, what is the current state of things then I think telehealth is better’ (Child C); ‘It depends on what it is. If you are being tested obviously in person but if it is just catching up then telehealth would be fine with me’ (Child I); ‘I think it depends on what it is for. If it is a bit more hard to explain or you need to see the child in person. Sometimes it is a bit difficult if you on a screen or on a phone but I think it just varies depending on the appointment’ (Child B).

While still having a positive experience in the telehealth appointment, some families where English was a second language preferred face‐to‐face appointment as it was easier for them to communicate in that context: ‘Overall it was positive but I have a little bit language barrier because I am not an English native speaker so I prefer face‐to‐face’ (Child K). Other families from a culturally and linguistically diverse background favoured a combination of face‐to‐face and telehealth appointments. There was no clear difference in the preference of appointment type between cultural and language backgrounds.

There was also no particular trend in the preference of appointment type based on the age of the child, diagnostic outcomes or assessment completed. Nearly half of the families reported that a combination of telehealth and face‐to‐face appointments was preferable, with the type of appointment dependent on the context of the consultation, availability and participants involved in the appointment.

#### 6.2.4 Areas of improvement

While most families did not offer suggestions for improving the telehealth experience, a few parents/carers commented that improving the quality of the telehealth platform and providing clearer instructions for navigating the telehealth appointments would be beneficial: ‘…as long as people have the information of what to do and how to do it prior… if it was in advance then step by step, go to this, click on this, log onto this or a link. I just think that being prepared in advance would help a lot especially for those that are not sure and they can jump on and figure it out…’ (Child B).

### 6.3 Staff focus group

#### 6.3.1 Advantages of the split service delivery model

##### Convenience

Staff members reported that some families appeared more comfortable with the telehealth appointments as they could remain at home and the reduction in hospital visits was perceived as convenient. The hybrid model was perceived to enable easier service access for families in rural and remote areas (see Figure 3).

##### Flexibility

Clinicians indicated that utilizing telehealth promotes flexibility in clinical practice, work location and use of time. Clinicians felt that they could more easily make appropriate arrangements to meet the needs of families: ‘People have been able to work from home to do the interviews and some feedbacks. Parents that aren't able to spend the whole day can join in for just important parts or relevant parts of the assessment’. Some staff also commented that the hybrid model allowed them to work from home if they had slight physical symptoms, or were required to isolate, therefore allowing appointments to continue.

##### Improved clinical service

Clinicians perceived that the use of telehealth increased the engagement of different stakeholders such as caseworkers and other healthcare professionals involved in the care of the child, school staff and parents: ‘It allows the dad to be more involved within the feedback session or if split families to be able to attend it, mum and dad in the same space to give feedback to both parents at the same time without being physically together. We have also had good success with foster parents and the caseworker online with us to give feedback. That was fantastic’. Clinicians felt that they had the opportunity to gather more information after the initial telehealth consultation and therefore were better prepared for the upcoming face‐to‐face appointment with the families: ‘some of the clinicians, particularly the doctors who take the history like that they might have two weeks between history and the appointment to contact schools’. Comprehensive assessment and better communication between the children's care providers can be achieved.

##### Improved assessment skills

Staff have noted that over time, there has been improvement to service delivery using the hybrid model of assessment. This has included staff realizing that they have adapted communication styles and assessment techniques when relating to families online, resulting in what has been perceived to be an increase in parent and child engagement during telehealth.

#### 6.3.2 Disadvantages of the hybrid service delivery model

##### Technical issues

Technology challenges from the hospital and family's end were reported to be a disadvantage of the hybrid delivery service model. Staff experienced different technical glitches when conducting telehealth appointments with families: ‘Often families were in poor internet connection areas. Our internet connection was quite poor as well’. They also reported that some families had bandwidth issues, ‘Even sometimes trying to get them on a mobile phone or a telephone could be difficult’, or that families were not financially able to upgrade their mobile data plan for more seamless telehealth appointments: ‘parents often ran out of money and then they didn't have access to the phone’.

##### Challenges to engage with families

Clinicians perceived that it is more difficult to get to know the child via telehealth, and that additional time is required to build rapport and trust with families when compared to face‐to‐face appointments. Reading body language and subtle behaviours of the child and family members was also more difficult with telehealth, resulting in challenges when building personal connection and providing emotional support: ‘I think we underestimate how much or how important that [rapport] is in building a relationship with our families and you know some of the gestures we use or the support we can offer them with a box of tissues or a warm word or stroke their arm or something. We can't do any of that to support them or even asking them questions and then becoming distressed so I miss that physical contact’. Distractions within the home could also affect rapport: ‘Parents were often at home stuck with kids not at school so there were a lot of other distractions going on at home’.

##### Team cohesiveness

Staff members reflected that since the hybrid model divides the assessment service into multiple appointments and team members may be connecting to appointments from different physical locations, there is an increase in disconnection amongst the group: ‘we check in with each other and [need] to find out what happened which we didn't have to do before’. Staff members reported that communication among the team has become more challenging under the hybrid service delivery model ‘now there is a lot more communication required from different members of the team’.

##### Longer waitlist and increased staff workload

The only appointments where clinicians felt an increase in workload and time were those involving interpreters. However, the administrative staff's workload did increase due to managing multiple bookings and cancellations. The current electronic booking systems did not enable them to alter appointments easily, resulting in more time and effort for rescheduling.

## 7 DISCUSSION

This study was a quality improvement project looking at the perceptions of parents/carers and staff on the use of telehealth and the development of a hybrid service delivery model at a metropolitan developmental assessment clinic by utilizing the PDSA cycle. This study was developed to understand the experience of families and staff while adapting to a hybrid model for the administration of complex developmental diagnostic assessments (plan). The hybrid model was implemented to ensure service continuity despite pandemic restrictions (do). The hybrid model provided families with an alternative means to gain an understanding of their child's needs and to empower them to advocate for appropriate early intervention funding and services to be initiated in a timely manner.

The new service delivery model was studied (do) to examine facilitators and barriers with the intention of improving future patient experience (study). The findings of this study demonstrated high acceptability of the integration of telehealth into the developmental diagnostic assessment service. The convenience and flexibility of telehealth appointments were commonly reported by families and staff. Families appreciated the continuation of services and support via telehealth during the COVID‐19 pandemic, whereas staff valued the application of telehealth to improve clinical service and professional skills.

The feedback from families and staff informed the refinement and development of this service delivery model within the clinic (act). Our findings highlighted the importance of preparing parents and children and managing parent expectations before the telehealth appointment. Accordingly, information sheets were given and preappointment phone calls were made to families to explain the clinician's goals within the telehealth appointment and how they would complement face‐to‐face appointments within the hybrid service delivery model. This study also informed the need to reduce the burden on administrative staff in implementing online processes; identified unwieldy booking systems; and used the hybrid model to create a greater variety of appointment types. This has increased the number of patients who can be fully assessed over a set period of time. As time progresses and with increased familiarity, systems will be further refined to increase the efficiency of administrative and clinical procedures.

Furthermore, our findings highlighted the need to increase staff proficiency and comfort levels using telehealth for developmental diagnostic assessment. The conversational aspects of the assessment process were identified as being more readily transferable to the telehealth environment, with more work needing to be done to determine when telehealth may provide an appropriate alternative option to face‐to‐face assessment while maintaining high fidelity and reliability over time. Hence, our clinicians have received additional training on diagnostic assessments that can be reliably conducted via telehealth. This is not only beneficial for overcoming pandemic‐related barriers but also for children and families living in rural and/or remote regions of Australia, where access to such services is limited.^34^

Due to the diagnostic complexity of the population who attend developmental services, there is no ‘one size fits all’ approach. While this study focussed on parental and staff perceptions and experiences, ongoing advancements in telehealth for direct developmental assessments will mean that more research is needed to explore online protocols for developmental and autism assessments; the profile of children and families who best suit these assessments; ways to ensure the reliability/validity of the assessments; and increase uptake by referrers and parents/carers. Accordingly, our research team has been developing new projects to further evaluate the acceptability of the telehealth diagnostic assessment and consumers' feedback on the assessment process. There is a need to explore the relationship between sociocultural, educational and practice‐related factors that influence comfort levels of clinicians and administrators towards the uptake, utilization and dissemination of telehealth in clinical practice.

### 7.1 Limitations

There are some limitations to this quality improvement project, which include the small sample size of parents/carers who completed the online telehealth questionnaire and interviews. A larger sample size would draw on a greater range of experiences. Those parents/carers who completed the questionnaire and were interviewed had adequate English skills to effectively communicate with the research team and no interpreter was used. Only those parents who opted for telehealth assessment were included in the study. The latter two factors reflect possible bias in the sampling and therefore may not identify the full range of issues and complexities faced by families from culturally and linguistically diverse groups and the broader population when engaging in telehealth services.

Additionally, the interviews with parents/carers were completed within a range of 1 week to 5 months post telehealth appointments. This variation and hence reliance on parent/carer memory could influence the quality and specificity of the information obtained.

## 8 CONCLUSION

The importance of being able to connect with and access health services during a time of public health restrictions forbidding prolonged face‐to‐face contact is reflected in the feedback received from both parents/carers and staff. In this study, factors that contributed to the successful delivery of telehealth services were identified by both parents/carers and clinicians. This mode of service delivery received high acceptance from both groups. The identification of barriers, such as technical issues, limited ability to build rapport between families/clinicians and limited opportunities for direct assessment of the child, provides clinicians with the opportunity to put strategies in place to reduce these barriers and further increase the success of telehealth within this clinical setting.

Further studies and research into effective means of directly observing and using standardized assessments via telehealth would be most valuable to increase the scope, validity and reliability of the use of telehealth within a paediatric diagnostic developmental service. There is also the potential to use telehealth for developmental screening programmes enabling families to access relevant services in a timely manner. With increased evidence supporting the equivalence of telehealth and face‐to‐face assessments, it is expected that uptake of telehealth by parents and clinicians will increase. Research should also obtain opinions of those who did not opt for telehealth to determine barriers to uptake of telehealth. Further research into understanding the factors affecting team cohesiveness and client/clinician interactions when working via telehealth would also facilitate improved telehealth service delivery, particularly within a complex clinical service, and result in a continuance of hybrid service delivery beyond the immediate COVID‐19 pandemic.

This study indicates that there are many positive aspects of delivering a service via telehealth, even within a complex paediatric setting. Even when public health restrictions forbidding prolonged social contact cease, there is a place for telehealth service delivery to provide the flexibility, convenience and cost‐saving advantages identified by both consumers and clinicians.

## AUTHOR CONTRIBUTIONS

All authors have made substantial contributions to the work. Esther Chan, Natalie Ong and Diana Barnett substantially contributed to the study conception, and all authors contributed to the design. Esther Chan, Natalie Ong and Diana Barnett contributed to the data collection, analysis and interpretation of data for the research findings. All authors reviewed drafts of the manuscript and revised the manuscript critically for important intellectual content. All authors have read and approved the final manuscript, and have agreed to the listing order for authors.

## CONFLICT OF INTEREST

The authors, Marie Antoinette Hodge, Suzi Drevensek, Marcia Williamsz and Natalie Silove, were involved in the staff focus group as participants.

## Data Availability

The data that support the findings of this study are available from the corresponding author, Esther Chan, upon reasonable request.
